# Sperm protein 17 targeting for epithelialovarian cancer treatment in the eraof modern immunoengineering

**DOI:** 10.1016/j.omto.2021.10.010

**Published:** 2021-10-28

**Authors:** Maria Poplawska, Dibyendu Dutta, Yichun Lee, Seah H. Lim

**Affiliations:** 1 Division of Hematology and Oncology, Department of Medicine, SUNY Downstate Health Sciences University, Brooklyn, NY, USA; 2 Division of Gynecologic Oncology, Department of Obstetrics and Gynecology, SUNY Downstate Health Sciences University, Brooklyn, NY, USA

**Keywords:** Sperm protein 17, tumor antigens, epithelial ovarian cancer, cell-based immunotherapy, immunoengineering

## Abstract

Due to the vague symptomatology of the disease and a lack of effective screening methods, most patients with epithelial ovarian cancer (EOC) present late in their disease. Despite advances in chemotherapeutic agents, the prognosis of these patients has uniformly been extremely poor. Although cisplatin-based chemotherapy regimens induce responses in most of these patients, the patients invariably experience disease progression or relapses. In an attempt to improve the treatment outcome using a different therapeutic approach, immunotherapy was investigated nearly 20 years ago. Many tumor antigens that are potentially suitable for specific immunotherapy were identified, and many immunotherapeutic approaches were attempted. However, although some responses were observed, the results from clinical studies were generally disappointing. Recent advances in immunoengineering and successes observed among patients treated for refractory/relapsed hematologic malignancies have rekindled the interest to revisit specific cellular immunotherapy in EOC. In this review, we provide the rationale for immunotherapy of EOC, discuss the results of some of the historical studies on the use of cellular immunotherapy in EOC, outline the principles of modern immunoengineering that could be applied to treat the disease, and propose the re-evaluation of the cancer-testis antigen, Sperm protein 17, for targeting by using modern immunoengineering technology.

## Graphical abstract

**Figure:**
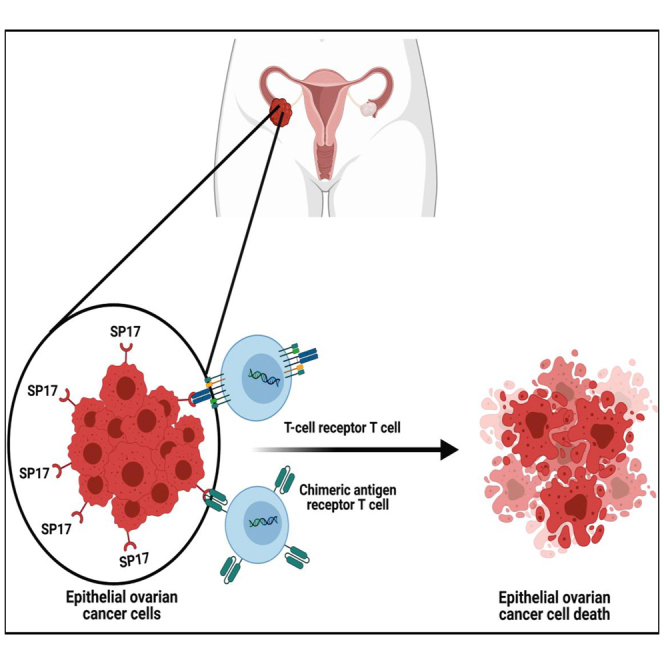

## Teaser

Sperm protein 17, a normal testicular protein aberrantly expressed in epithelial ovarian cancer, is a highly immunogenic tumor antigen discovered more than 20 years ago. The authors provide a review of how this antigen could be revisited for modern immunotherapy of ovarian cancer in the era of immunoengineering.

## Introduction

Epithelial ovarian cancer (EOC) remains one of the commonest causes of cancer-related deaths in women in the 21st century. In the United States, there were approximately 235,000 EOC patients in 2018.^1^ It is estimated that there will be 21,410 new cases, with 13,770 deaths, in the United States in 2021.^1^ Although EOC is potentially a curable disease in its early stages, due to the generally vague and nonspecific symptoms and lack of effective screening strategies, most patients present late in their disease when metastasis has already occurred. Despite the advances in modern chemotherapeutic agents and personalized medicine, a significant inroad into the treatment of EOC has not been made. Cytoreduction through primary surgery followed by platinum-based chemotherapy has remained the first-line treatment for women with newly diagnosed EOC,^2^ and this has not changed significantly in the last three decades. While a high proportion of patients will respond to the combination of surgical debulking followed by adjuvant chemotherapy with carboplatin and paclitaxel, disease recurrence/progression invariably occurs.^2^

New approaches for advanced EOC in the past decade include antiangiogenic agents^3^, ^4^, ^5^ and poly(ADP-ribose) polymerase (PARP) inhibitors.^6^^,^^7^ The most commonly used and well-studied antiangiogenic agent in the treatment of EOC is bevacizumab. However, as reported in ICON-7^8^ and GOG 281,^9^ bevacizumab added to platinum-based chemotherapy did not increase overall survival (OS) in the study populations as a whole but did show a significant difference in OS in a predefined subgroup of patients with poor-prognosis disease in ICON-7 and stage IV disease in GOG 218. PARP inhibitors, on the other hand, have shown meaningfully prolonged progression-free survival (PFS) for the first time after decades of studying different chemotherapy approaches.^10^ The well-studied PARP inhibitors for the treatment of high-grade serous epithelial ovarian, fallopian tube, or primary peritoneal cancer include olaparib, rucaparib, niraparib, and veliparib. Most of them are approved by the Food and Drug Administration to be used for first- or second-line maintenance therapy after cytotoxic chemotherapy or as the single agent for third- or fourth-line therapy. Optimal use of these newer agents remains to be determined, but at present the clinical benefits in OS are modest. The lack of successes with the currently available chemotherapeutic agents suggests that other approaches are much needed.

In this review, we discuss briefly the principles and history of immunotherapy in EOC, dissect Sperm protein 17 (Sp17) as a target for immune targeting, and examine how the current advances in immunoengineering might be exploited to utilize Sp17 for cell-based therapy of EOC.

## Principles and history of immunotherapy in EOC

Immunotherapy has always been an attractive cancer therapeutic option, since it is not only more specific but also kills tumor cells via molecular mechanisms different from those induced by chemotherapeutic agents. It may, therefore, be used to either circumvent chemoresistance or in conjunction with chemotherapy to augment the cytotoxicity. Various clinical and laboratory data support the applicability of immunotherapy in EOC.

Both extracellular and intracellular tumor antigens have been demonstrated in ovarian cancer. Examples of these antigens are shown in Table 1.^11^, ^12^, ^13^, ^14^, ^15^, ^16^, ^17^, ^18^, ^19^, ^20^, ^21^, ^22^, ^23^, ^24^, ^25^, ^26^, ^27^ Most of these antigens arise due to either overexpression or aberrant expression of normal cellular proteins. Extracellular antigens include Sp17,^18^ MUC1,^11^ and mesothelin.^14^ Intracellular tumor antigens in ovarian cancer cells have also been demonstrated in various studies, including the MAGE family of antigens,^16^ NY-ESO1,^17^ and LACE-1.^17^ Other antigens including neoantigens^24^, ^25^, ^26^, ^27^ may have arisen from random mutations associated with neoplastic process, although genomic analysis of EOC has found the tumor cells to harbor low mutational burden.^28^

**Table 1:** Tumor antigens in epithelial ovarian cancer

Class	Antigens	Comments
Overexpressed normal cellular antigens	MUC1	these are normal cellular proteins that are overexpressed on EOC cells. Antigen-reactive T cells within the host immune repertoire are expected to be of low affinity for the tumor cells, since any high-affinity T cells would have been deleted from the immune repertoire during development to avoid autoimmunity
	Wilms’ tumor 1 (WT1)	
	Telomerase	
	Mesothelin	
	Erb	
Cancer-testis antigens (CTA)	MAGE family	these are normal testicular proteins that are aberrantly expressed by EOC cells. They are generally highly immunogenic because testes are immune sanctuary sites, hence high-affinity antigen-reactive T cells are not deleted by the thymus during development. Furthermore, being whole proteins, immune responses tend to be polyclonal, directed at more than one epitope within the antigens, providing high *in vivo* effector/target ratios. Even though CTA are testicular-restricted proteins, highly sensitive qPCR has detected leakage of expression of some of these genes, at low levels, in normal tissues. Their expression in tumor cells is believed to be mediated, in most cases, by changes in the methylation status of the gene during carcinogenesis
	NY-ES O 1	
	LACE-1	
	Sp17	
	AKAP3	
	AKAP4	
	GAGE-1/2	
	PRAME	
	TRAG-3	
Neoantigens	mutated KRas	these antigens arise due to point mutations that cause amino acid substitutions. Being neoantigens, they are highly immunogenic. However, unless there are numerous mutations within the genes, immune responses against the neoantigens will be monoclonal, directed at only one single epitope, restricting the resulting *in vivo* effector/target ratios
	mutated p53	
	mutated Erb	
	mutated ARID1a	

EOC cells are susceptible to the cytotoxic machinery of T cells. Peripheral blood lymphocytes primed to MUC1,^29^ MAGE,^30^^,^^31^ Sp17,^32^ and mesothelin^33^ have demonstrated the ability to lyse EOC cells. Similarly, susceptibility of EOC cells to killing by T cells has been demonstrated in mouse models of EOC.^34^^,^^35^ Clinically, the correlation between the intensity of tumor-infiltrating lymphocytes (TILs) and clinical outcomes^36^, ^37^, ^38^ further supports this notion. Clinical responses were observed in EOC patients treated with TILs expanded *ex vivo*.^39^, ^40^, ^41^, ^42^

Since ovarian tumor cells harbor immunogenic antigens and tumor-reactive T cells are present in the immune repertoire of the autologous hosts, harnessing the host *de novo* antitumor immune responses through immunotherapy is an attractive option. Unfortunately, the results from cell-based immunotherapy in EOC have thus far been uniformly disappointing (Table 2).^39^, ^40^, ^41^, ^42^, ^43^, ^44^, ^45^, ^46^ One of the major reasons for the lack of successes is an immune repertoire that has been contracted severely by chemotherapy, rendering the host response to be unable to mount an efficient effector/target ratio *in vivo*. This notion is clearly seen in the clinical use of immune checkpoint inhibitors in EOC patients. Due to low tumor mutational burden (17) and thus a low number of neoantigen-reactive T cells, the response rate has been uniformly disappointing. Advances in immunoengineering provide a mean to increase the effector/target ratio. However, this approach is applicable only for known antigens and not for unidentified neoantigens that arise from random genomic mutation in EOC.

**Table 2:** Samples of clinical studies of cell-based immunotherapy in epithelial ovarian cancer

Authors	No. of patients (n)	Intervention	Outcome	Comments
Kershaw et al.^43^	8 in cohort 1; 6 in cohort 2	T cells with reactivity against the ovarian cancer–associated antigen α-folate receptor (FR) were generated from autologous T cells with a chimeric gene incorporating an anti-FR single-chain antibody linked to the signaling domain of the Fc receptor γ chain. Eight patients in cohort 1 received a dose escalation of T cells in combination with high-dose interleukin-2, and six patients in cohort 2 received dual-specific T cells (reactive with both FR and allogeneic cells) followed by immunization with allogeneic peripheral blood mononuclear cells	no response was observed. ^111^In-labeled adoptively transferred T cells in cohort 1 did not show any tumor localization except in one patient where some signals were detected in the peritoneal deposit	phase 1 study in which all 14 patients had metastatic disease and had undergone surgical debulking and combination chemotherapy
Haas et al.^44^	15 patients in total: 6 in cohort 1; 3 each in cohorts 2, 3, and 4	CAR was directed at mesothelin and contained CD3z and 4-1BB in the lentiviral construct. Cohorts 1 and 2 patients received 1–3 × 10^7^ m^2^ and cohorts 3 and 4 patients received 1–3 × 10^8^/m^2^ CAR-T cells. Patients in cohorts 2 and 4 also received lymphodepletion with cyclophosphamide, 1.5 g/m^2^ 3 ± 1 day prior to CAR-T infusion	stable disease was observed in 11/15 patients on day 28	phase 1 study that included 5 patients each, with ovarian cancer, malignant pleural mesothelioma, and pancreatic cancer
Aoki et al.^39^	17 patients with advanced/recurrent EOC	7 patients received TILs following one dose of cyclophosphamide (group A) and 10 received alternating cisplatin-containing regimen and TILs (group B)	1 CR and 4 PR in group A, and 7 CR and 2 PR in group B	responses in both primary and metastatic lesions lasted 3–5 months in group A
Fujita et al.^40^	13	all patients were treated in the adjuvant setting after surgical debulking and cisplatin regimen. Each received >1 × 10^9^ TILs	3-year DFS was 81.2% and OS 100%	compared with a control group of 11 patients, DFS and OS were significantly prolonged
Freedman et al.^41^	8 patients with advanced EOC refractory to platinum-based chemotherapy	all patients received 10^10^–10^11^ TILs intraperitoneally and recombinant interleukin-2. One patient received two doses of TILs	no measurable response was observed in any of the patients	pilot study to evaluate feasibility and clinical response of intraperitoneal TILs
Liu et al.^45^	92	all patients were treated in the adjuvant setting following surgical debulking and 6–8 cycles of carboplatin/paclitaxel; 46 patients were treated with monthly CIK cells and 46 in the control group with observation only	PFS was 37.7 months in the treatment group compared with 22.2 months in the control group (p = 0.004). OS was not improved	paired study of monthly maintenance CIK cells
Pedersen et al.^42^	6	all patients had platinum-resistant progressive metastatic EOC. They all received TILs followed by rIL-2	4 patients showed stable disease for 3 months and 2 for 5 months	high number of infused TILs expressed LAG-3 and PD-1
Wright et al.^46^	7	all patients had recurrent EOC confined to the peritoneal cavity. They received peripheral blood lymphocytes stimulated with MUC1 intraperitoneally	1 patient attained CR	no significant reduction in serum CA125

CR, complete response; PR, partial response; DFS, disease-free survival; OS, overall survival; PFS, progression-free survival; TILs, tumor-infiltrating lymphocytes; CIK, cytokine-induced killer; rIL-2, recombinant interleukin-2; MUC1, Mucin 1; LAG-3, lymphocyte activation gene 3; PD-1, programmed cell death protein 1; CA125, carbohydrate antigen 125.

## Sperm protein 17 as a target for immunotherapy

### Sp17 gene and protein

Human *Sp17* gene, located on chromosome 11q24.2, encodes a highly conserved antigenic protein of 17.4 kDa molecular weight expressed in spermatozoa and is involved in acrosome reactions during fertilization.^47^ Immunohistochemistry on tissue arrays and RT-PCR of a panel of tissue RNA^48^ showed Sp17 to be restricted in its normal tissue distributions, being expressed primarily in normal testes. Immunotherapy targeting Sp17 will, therefore, be more specific and less toxic.

Sp17 is a highly immunogenic protein. Many vasectomized normal males develop autoantibodies against Sp17.^49^ It was, therefore, the target of investigation as an immunocontraceptive.^50^ Sp17 is primarily an extracellular protein and promotes heparan sulfate-mediated cell-cell adhesion.^51^^,^^52^ Although it is an extracellular protein, Sp17 undergoes modulation and is shed into the serum of cancer patients.^53^

### Sp17 as a tumor antigen

Sp17 was first identified in our laboratory as an aberrantly expressed tumor antigen in multiple myeloma.^54^ It has since been found to be expressed in non-Hodgkin's lymphoma^55^ and non-small cell lung cancer.^56^ Interestingly, despite being a normal testicular protein, Sp17 expression on tumor cells does not appear to be gender biased. In fact, Sp17 is expressed in high proportions of patients with gynecologic cancers such as ovarian cancer, endometrial cancer, and cervical cancer (Table 3).^18^^,^^54^, ^55^, ^56^, ^57^, ^58^, ^59^, ^60^, ^61^, ^62^ In these conditions, Sp17 was not only expressed at the mRNA level but also at the protein level, arguing for a possible function of the protein in tumor cells. Sp17 expression is correlated with chemoresistance in clear-cell adenocarcinoma.^63^ It also has roles in metastasis and drug resistance; for example, EOC with high Sp17 expression shows resistance to paclitaxel,^64^ the first line of EOC treatment. EOC cells with enhanced Sp17 expression demonstrate increased migration.^65^ Progressive tumor growth by ID8 cells in mice is dependent on Sp17 expression.^66^ Immunotherapy targeting Sp17 is, therefore, attractive and may be used to eradicate the chemoresistance clones to augment the efficacy of chemotherapy.

**Table 3:** Expression of Sperm protein 17 in tumor specimens

Authors	Tumor type	Comments
Lim et al.^54^	multiple myeloma	detected by RT-PCR and western blot analysis in 12/47 (26%) of specimens
Straughn et al.^18^	ovarian cancer	detected by RT-PCR, northern blot analysis, western blot analysis, and immunohistochemistry in 7/11 (64%) of papillary serous or mixed, 4/8 (50%) endometrioid, 1/1 primary peritoneal carcinoma, 2/2 clear cell, 3/3 ovarian unspecified. Overall, 17/25 (68%) specimens
Liggins et al.^55^	diffuse large B cell lymphoma	detected by RT-PCR, western blot analysis, and immunohistochemistry in 6/11 (55%) specimens
Zhou et al.^57^	invasive breast cancer	detected by RT-PCR and immunohistochemistry in 27/100 (27%) specimens. Sp17 expression correlated with lymph node metastasis
Mirandola et al.^58^	invasive breast cancer	detected by immunohistochemistry in 10/22 (45%) invasive breast cancers and 17/36 (47%) triple-negative breast cancers
Li et al.^59^	endometrial and cervical cancer	detected by immunohistochemistry in 33/50 (66%) endometrial cancers and 19/31 (61%) cervical cancers
Grizzi et al.^60^	neuroectodermal tumors	detected by immunohistochemistry in 6/28 (21%) tumors
Gupta et al.^61^	esophageal squamous cell cancer	detected by RT-PCR in 26/30 (86%) and by immunohistochemistry in 60/80 (75%) specimens
Xia et al.^62^	hepatocellular cancer	detected by immunohistochemistry in 36/45 (80%) specimens
Mirandola et al.^56^	non-small cell lung cancer	detected by RT-PCR and immunocytochemistry in 16/40 (40%) specimens

We and others have previously demonstrated the ability to generate Sp17-specific human leukocyte antigen (HLA) class I-restricted cytotoxic T cells from patients with various cancer types,^32^^,^^67^^,^^68^ including ovarian cancer. These cytotoxic T cells were able to kill fresh autologous ovarian tumor cells.^32^ T cell epitopes restricted by HLA-A1 and HLA-A2 have been identified.^68^^,^^69^ These works suggest that, despite being a surface antigen, Sp17 protein is also processed and presented in conjunction with the major histocompatibility complex (MHC). Furthermore, they also clearly demonstrate that despite the presence of active cancer and previous chemotherapy, Sp17-reactive T cells were still present in the immune repertoire of these patients. However, while molecules of MHC I expression are retained, Sp17-positive tumor cells have lower expression of MHC II molecules along with an increase in STAT3.^66^ STAT3 overexpression is associated with invasive and metastatic properties of EOC cells.^70^

### Regulation of Sp17 expression

Unless the tumor antigen is a driver protein needed for tumor cell survival, progressive downregulation of a tumor antigen gene often occurs. This is a form of immune tumor escape mechanism and poses one of the biggest obstacles to successful immunotherapy. Although there is currently no evidence supporting progressive downregulation of Sp17 expression, Sp17 is heterogeneously expressed within individual tumor specimens,^18^ suggesting the risk of selecting for Sp17-negative tumor clones. Sp17 gene expression is regulated through promoter methylation^71^ and its expression may be upregulated by DNA-hypomethylating agents such as 5-azacytidine,^71^ providing a means to circumvent the obstacle due to antigen downregulation and heterogeneity of the antigen.

## Sp17-targeted immunotherapy in the era of modern immunoengineering

Sp17 appears to be an ideal tumor target for modern immunotherapy of EOC for the following reasons: (1) it is expressed on the surface of a high proportion of EOC cells; (2) it is processed and presented by the MHC molecules on the surface of EOC cells; (3) it shows restricted normal tissue distribution; and (4) its expression may be regulated pharmacologically. Being a surface antigen, Sp17 may be amenable to specific antibody targeting. However, Sp17 monoclonal antibodies have not been tried in the clinic and the feasibility of this approach remains to be determined. Anti-Sp17 monoclonal antibody was found to have some antibody-dependent cellular cytotoxicity (ADCC) and complement-dependent cytotoxicity *in vitro* against Sp17-expressing ovarian cancer cells.^72^ Potential obstacles to its successful use include the presence of shed Sp17 protein in the serum of these patients.^53^ However, it remains to be determined whether the functions of ADCC remain intact and efficient in patients with advanced EOC.

**Figure 1:**
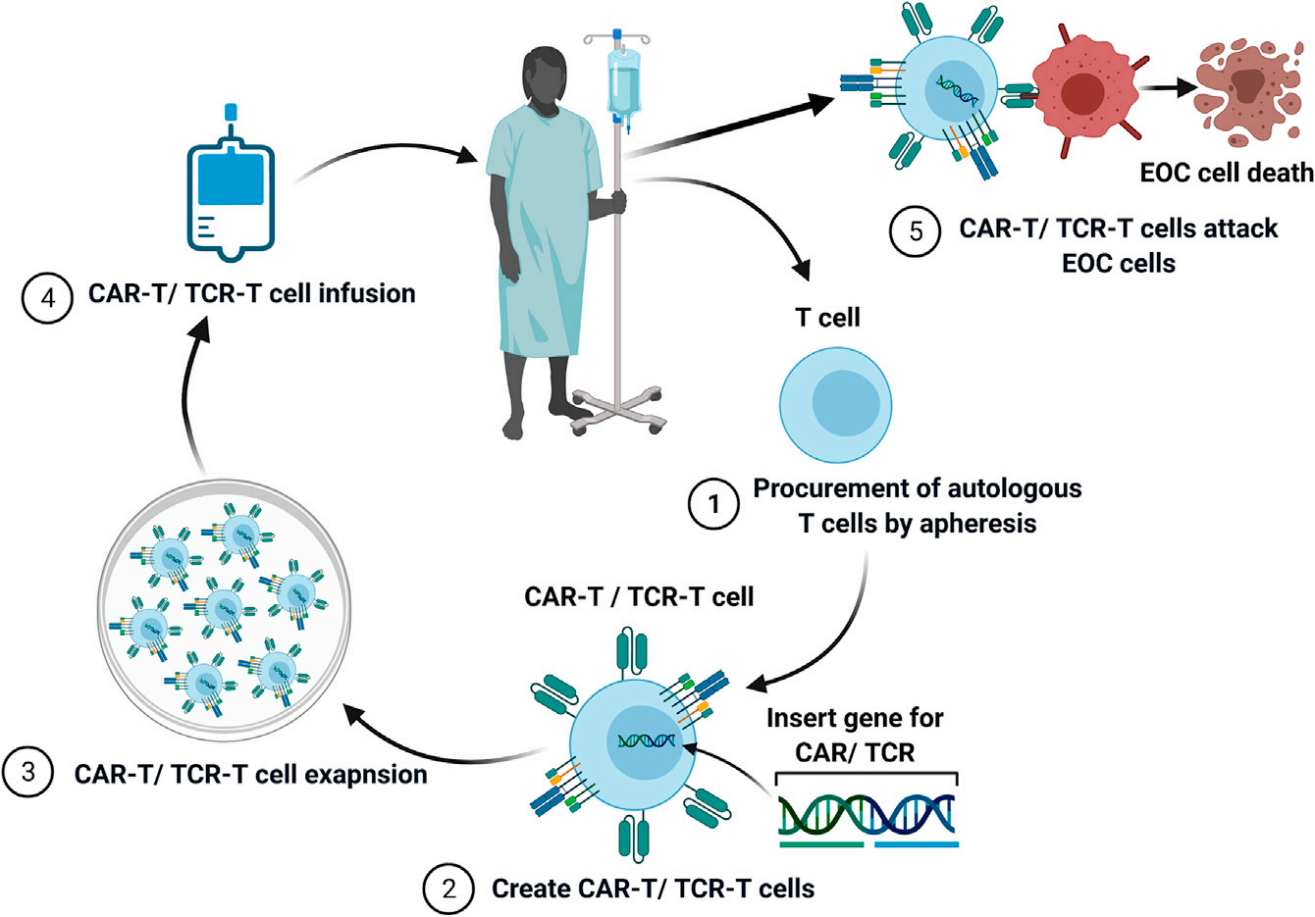
Schematic illustration of immunoengineering of CAR-T or TCR-T cells for treating patients with epithelial ovarian cancer (1) Autologous CD3^+^ T cells first are procured by apheresis from the patient. (2) T cells are genetically modified, most commonly using a lentiviral construct, to express chimeric antigen receptors (CARs) or engineered T cell receptors (TCRs). (3) CAR-T or TCR-T cells are expanded *ex vivo* to obtain required number. (4) Engineered T cells are infused into the same EOC patient to lyse EOC cells. Patients are commonly given chemotherapy via fludarabine and/or cyclophosphamide to lymphodeplete the immune repertoire prior to infusion of the engineered T cells. Lymphodepletion facilitates the homeostatic expansion of the infused engineered T cells.

On the other hand, data showing the susceptibility of ovarian tumor cells to T cells would support the use of T cell immunotherapy for EOC. In mice injected with ID8 ovarian cancer cells, coadministration of recombinant Sp17 and CpG oligonucleotides every 30 days for a total of ten doses prevented ovarian tumor formation for up to 300 days with a survival rate of 77% and 80% in prophylactic and therapeutic groups, respectively.^73^ It generated strong immune response evident by an increase in T-helper 17 cells, increases in tumor necrosis factor α, interferon-γ, and granulocyte-macrophage colony-stimulating factor in serum, and a decrease in regulatory T cells. Oral coadministration of M cell-targeted microparticles loaded with T and B cell immune-dominant region of recombinant Sp17 and CpG oligonucleotide triggered humoral and cellular immune responses in mice injected with ID8 ovarian cancer cells and retarded ovarian tumor growth.^74^ Human anti-Sp17 monoclonal cytotoxic T lymphocytes, obtained either from advanced ovarian cancer patients or from healthy donors, directly migrated to tumor sites and eradicated the tumor in mice injected with human ovarian cancer cells.^75^ Clinically, dendritic cells pulsed with the Sp17 recombinant protein were administered to one patient with EOC.^76^ Although no obvious clinical benefits were observed in this patient, there were transient drops in serum CA125. The transient drop in the tumor marker most likely reflects the low *in vivo* effector/target ratio generated by the dendritic cells within a very restricted immune repertoire. A modern immunoengineering approach would be an ideal platform for a cell-based immunotherapy for EOC targeting Sp17. Two approaches are currently available, chimeric antigen receptor T (CAR-T) cells or T cell receptor T (TCR-T) cells. Both approaches involve the procurement of autologous T cells by apheresis, followed by *ex vivo* expansion and genetic modification of the T cells in the laboratory to become either CAR-T or TCR-T, before the T cells are reinfused back into the patients (Figure 1). The *ex vivo* expansion will provide a means to increase the effector/target ratio following infusion of the genetically modified T cells.

### Chimeric antigen receptor T cells

CAR-T cells are autologous peripheral blood T cells engineered to display antigen-binding fragments of a specific antibody fused to intracellular T cell signaling domains. Human antibody variable fragments directed at Sp17 may be obtained through immunizing transgenic mice carrying the human immunoglobulin genes. CAR-T has produced encouraging clinical results in those with refractory B cell acute lymphoblastic leukemia,^77^ B cell non-Hodgkin's lymphoma,^78^ and multiple myeloma.^79^ CAR-T in hematologic malignancies have thus far relied primarily on targeting differentiation antigens to avoid potential antigen downregulation as a form of tumor escape from immunosurveillance.

**Figure 2:**
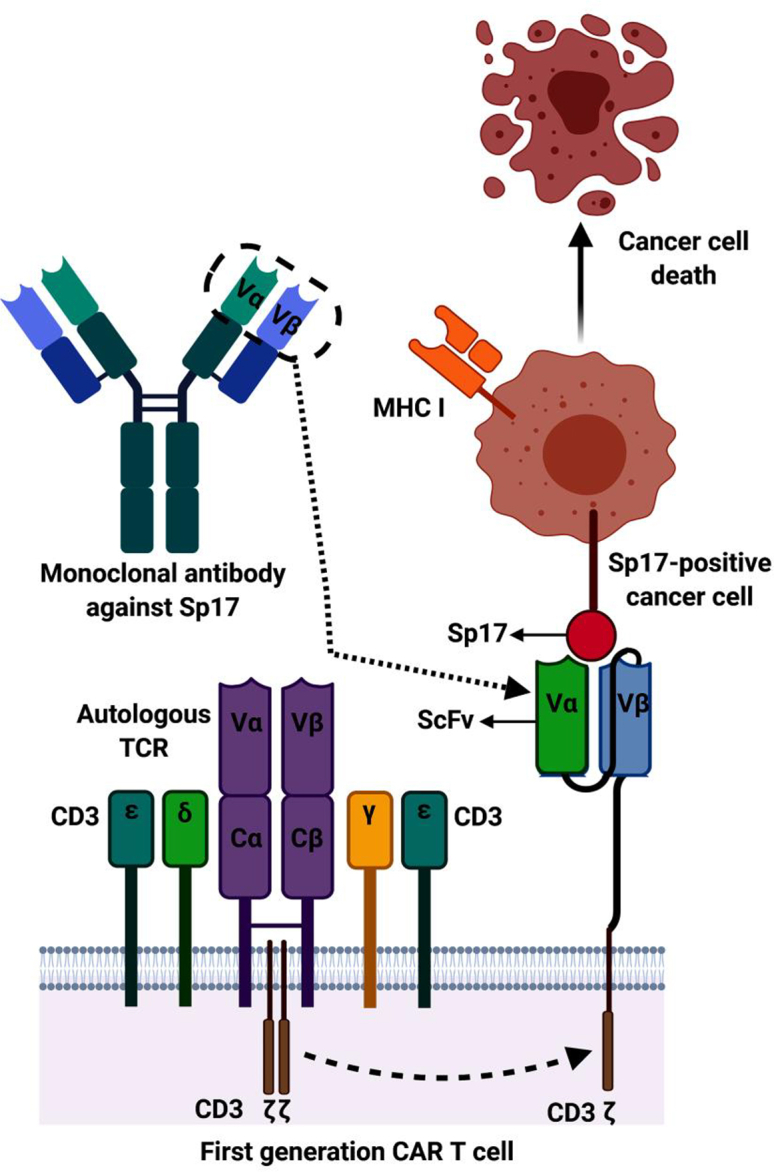
CARs are fusion protein receptors consisting of an extracellular single-chain fragment of variable regions (ScFv) derived from Sp17 monoclonal antibody This is most commonly accomplished through cloning of the immunoglobulin variable genes, v genes (VH and VL), encoding the Sp17 monoclonal antibodies. These v genes are connected through a linker and are cloned into a viral vector encoding the transmembrane CD3ζ, derived from T cells, to mediate CAR-T activation. Sp17-specific ScFv binds to EOC cells expressing surface Sp17 protein and mediates cell death through T cell cytotoxic mechanisms.

In EOC, there no suitable differentiation antigens have been identified so far. Sp17 is, therefore, a suitable alternative. CAR-T cells targeting Sp17 may be engineered to bind either the intact surface Sp17 or the HLA class I/Sp17 peptide complex (Figure 2) on EOC cells. Unlike other tumor antigens, Sp17 expression can be upregulated using DNA-hypomethylating agents.^71^ Intermittent upregulation of Sp17 expression may be sufficient to prevent tumor immunosurveillance escape and provide comparable efficacy with CAR-T that is directed at a differentiation antigen. The presence of both surface antigens and HLA class I/tumor peptide complex on the surface provides the opportunity for dual CAR-T therapy using a combination of CAR-T directed at the intact surface Sp17 protein and the HLA class I/tumor peptide complex to improve the efficacy of tumor cell killing and heterogeneity of antigen expression.

### T cell receptor T cells

**Figure 3:**
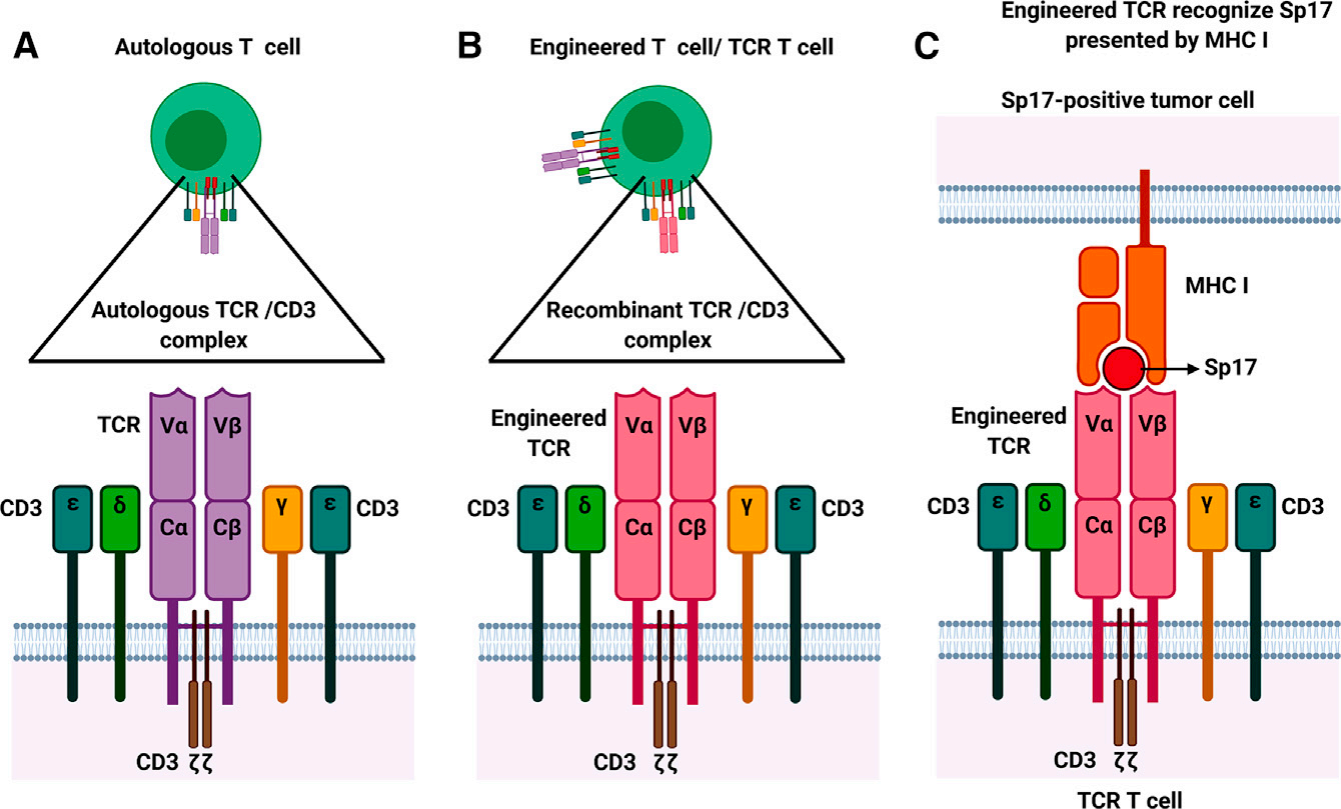
Schematic diagram of TCR T cell structure (A) The TCR of an autologous T cell is a heterodimer surrounded by CD3 chains. It recognizes intracellular antigens presented by the MHC molecules to initiate cell death. (B) The autologous TCR-T is genetically engineered by cloning of the TCR variable genes (Vα and Vβ) expressed by Sp17-reactive T cells to a viral vector expressing the TCR C genes (Cα and Cβ) linked to the transmembrane CD3ζ and transduced into autologous T cells. (C) Sp17 immunogenic peptides presented on the EOC cell surface in association with the MHC I molecules is recognized by TCR-T cells to initiate cell death.

Cell-based immunotherapy targeting Sp17 may also be accomplished by TCR-T cells, which are autologous peripheral blood T cells engineered to display antigen-binding fragments of a specific TCR fused to intracellular T cell signaling domains (Figure 3). Successes in various laboratories^32^^,^^67^^,^^68^ to generate cytotoxic T cells directed against Sp17 suggest that this could be readily accomplished by cloning TCR from the cytotoxic T cell lines generated. TCR-T cells are currently being explored in various tumor types.^80^, ^81^, ^82^, ^83^ However, since TCR does not undergo hypermutation and affinity maturation like an antibody, the affinity of TCR-T cells for their targets will invariably be considerably lower than those of CAR-T.

### Enhancing the efficacy of cell-based immunotherapy targeting Sp17

**Figure 4:**
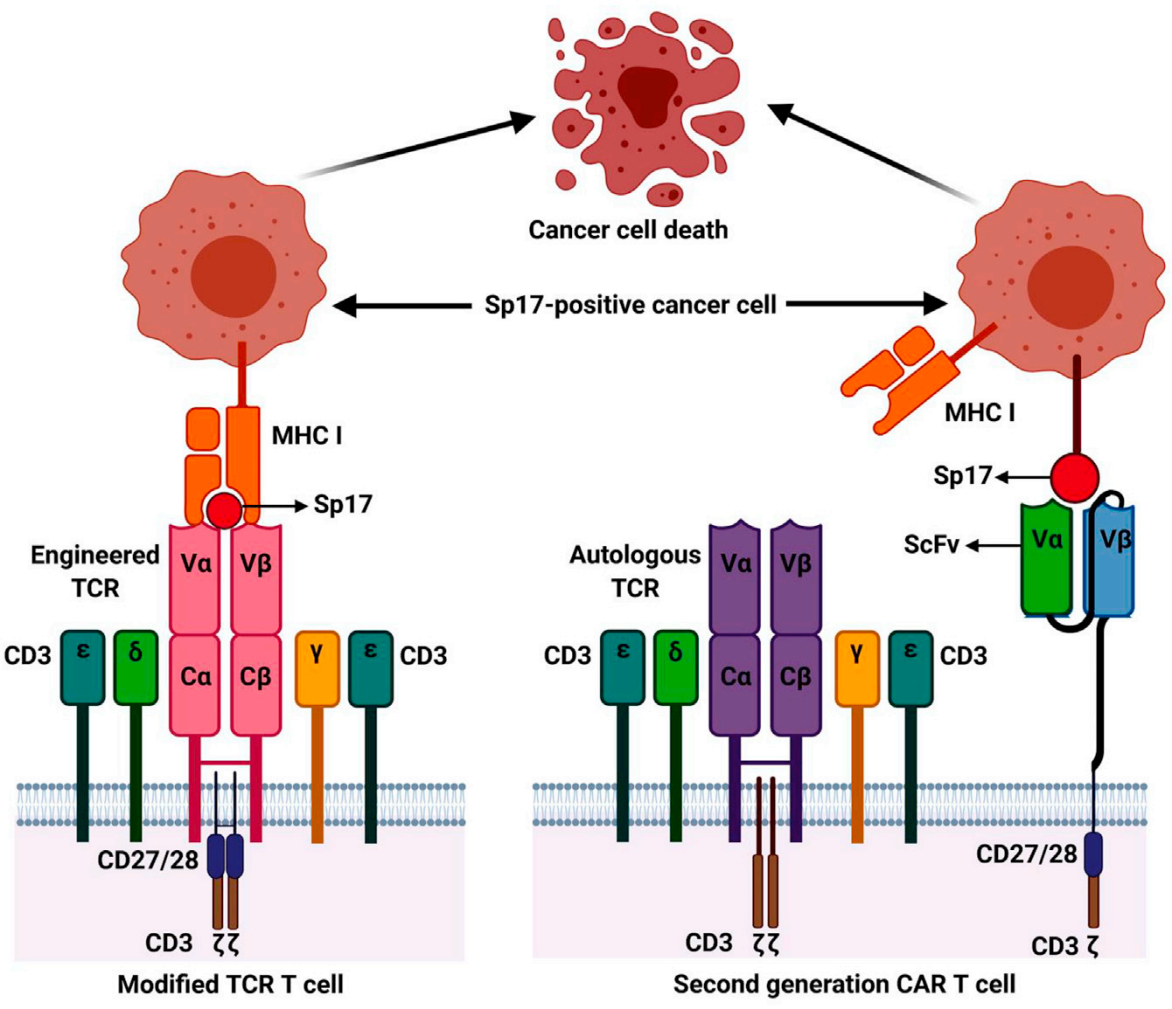
To enhance the efficacy of the TCR-T cell and CAR-T cell mediated immune response, the costimulatory domains of CD27 or CD28 are incorporated into the viral construct for either TCR-T cells or second-generation CAR-T cells

The efficacy of CAR-T cells and TCR-T cells may also be enhanced if the antigen-binding fragment is linked to the T cell costimulatory domains such as CD27^84^ or CD28^85^ (Figure 4). It may also be possible to improve the efficacy of the infused CAR-T or TCR-T cells by using them in combination with immune checkpoint inhibitors or sequential administration of chemotherapy. Other approaches to enhance the efficacy of cell-based immunotherapy for solid tumors include enhancing tumor cell apoptosis via iCaspase-9 release^86^ and strategies to overcome the adverse immunologic conditions induced by cytokines, such as transforming growth factors that impair T cell functions in the tumor microenvironment.^87^

## Conclusions

Recent advances in immunoengineering have rekindled interest in cell-based immunotherapy in EOC. It is expected that increasing efforts will be made to revisit the tumor antigens previously identified in solid tumors, especially cancer-testis antigens. However, although cell-based immunotherapy has transformed the treatment paradigm in a number of hematologic malignancies, it has so far not made significant inroads into solid tumors. Tumor microenvironments associated with solid tumors, such as malignant ascites and various immunosuppressive cytokines associated with EOC,^87^ will likely be the main obstacles impairing the efficacy of cell-based immunotherapy. Although there are many tumor antigens that may be suitable for targeting, in addition to enhancing T cell activation and upregulating antigen expression pharmacologically, successful cell-based immunotherapy for EOC may need to incorporate strategies that include sequential chemotherapy and methods to overcome the deleterious tumor microenvironmental cytokines.
